# Supplementary figures and images for: Phylogenetic and Comparative Analysis of Cryptochironomus, Demicryptochironomus and Harnischia Inferred from Mitogenomes (Diptera: Chironomidae)
Source: Insects. 2024 Aug 26;15(9):642. doi: 10.3390/insects15090642 (PMC11432083; doi:10.3390/insects15090642)

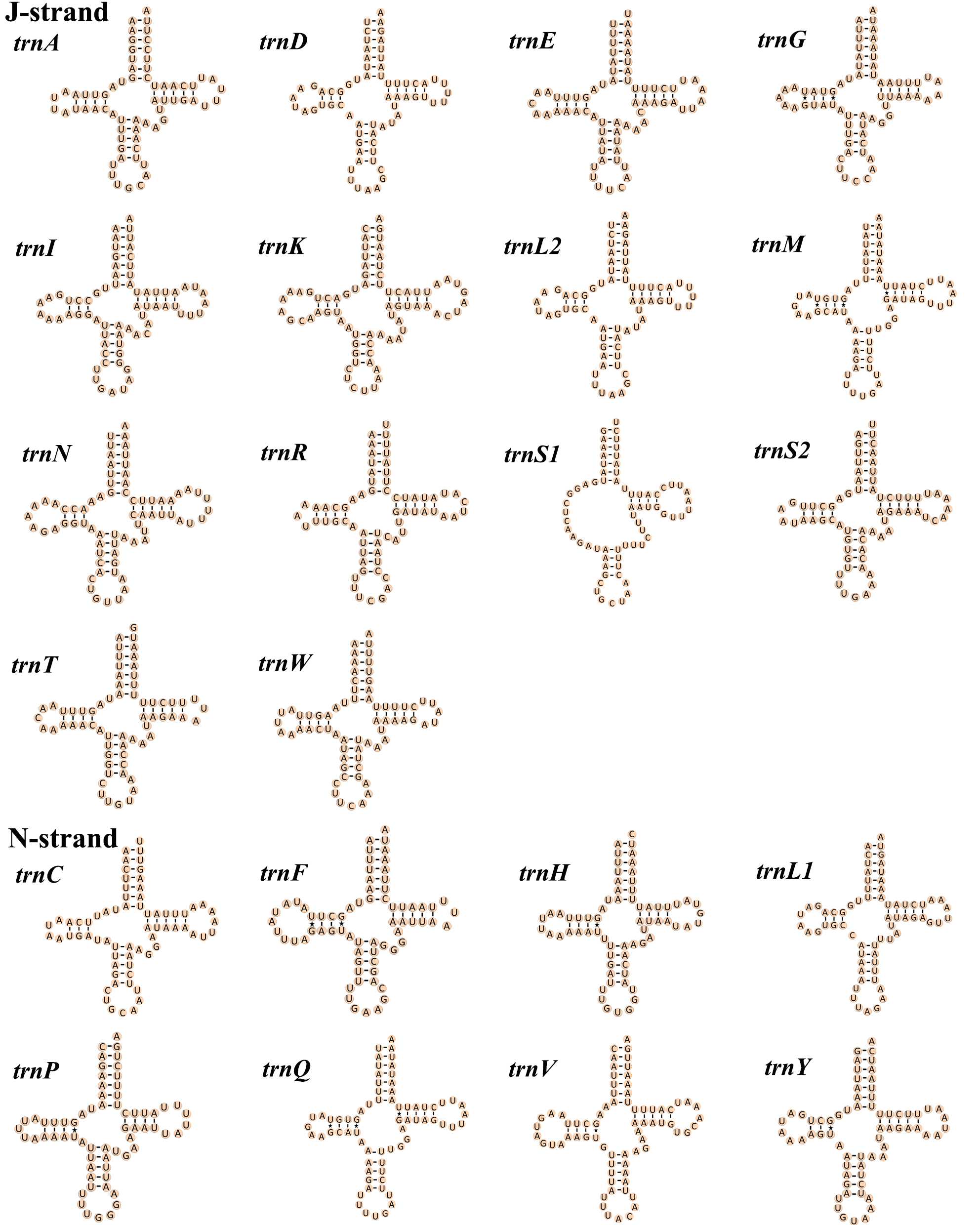

Supplement: Supplementary file 1 [file insects-15-00642-s001.zip › S1 Cryptochironomus maculus.jpg]

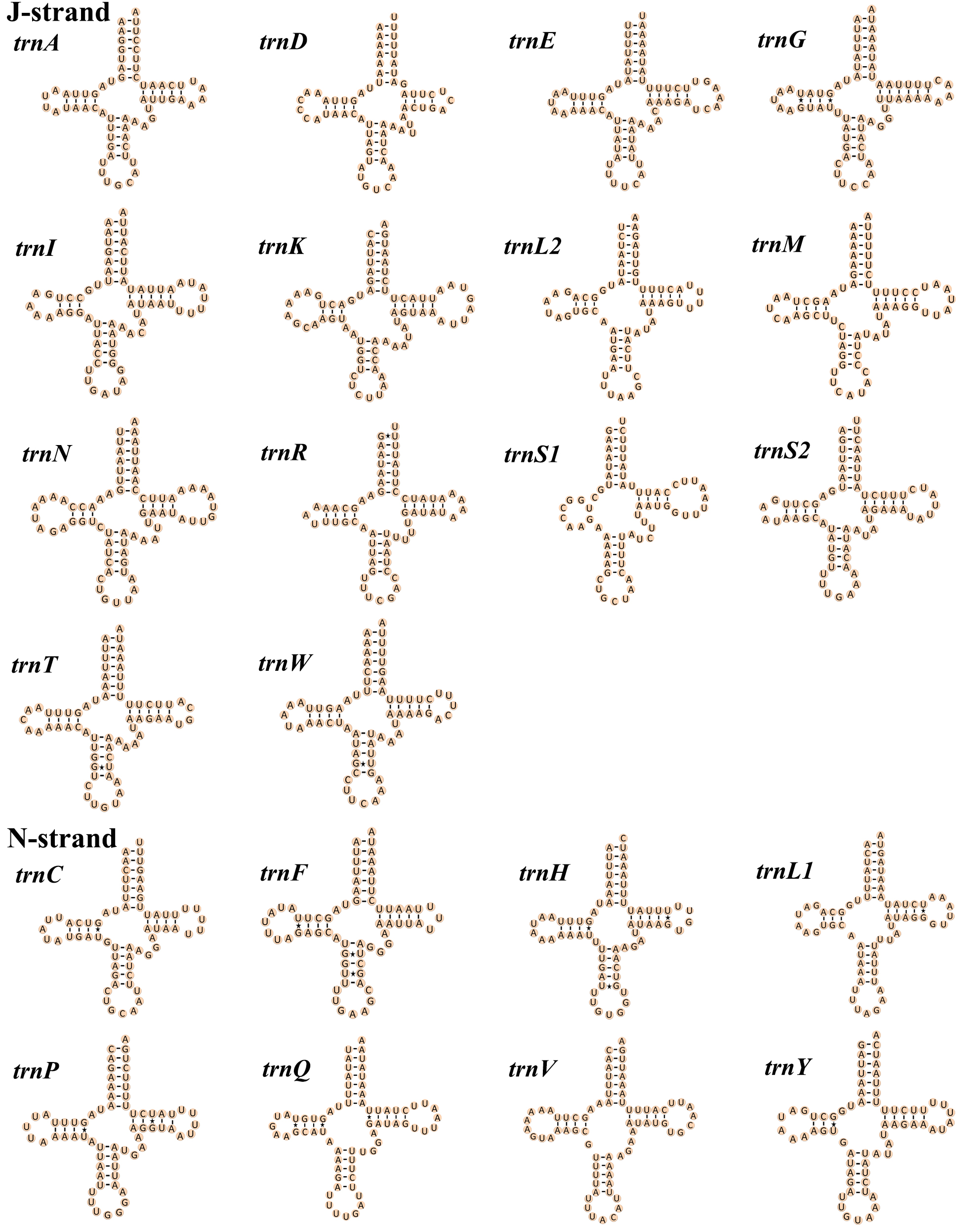

Supplement: Supplementary file 1 [file insects-15-00642-s001.zip › S10 Cladopelma virescens.jpg]

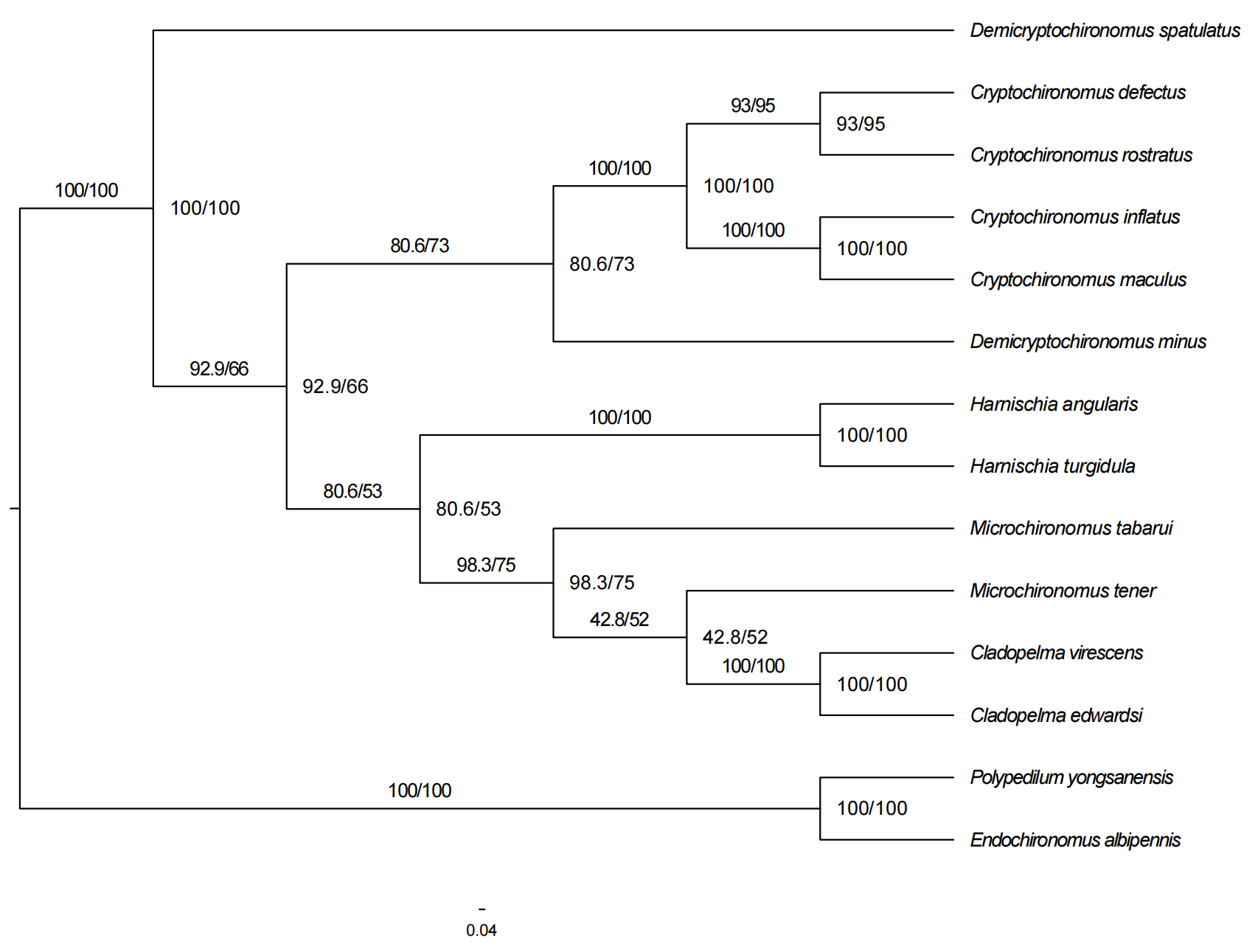

Supplement: Supplementary file 1 [file insects-15-00642-s001.zip › S11.tif]

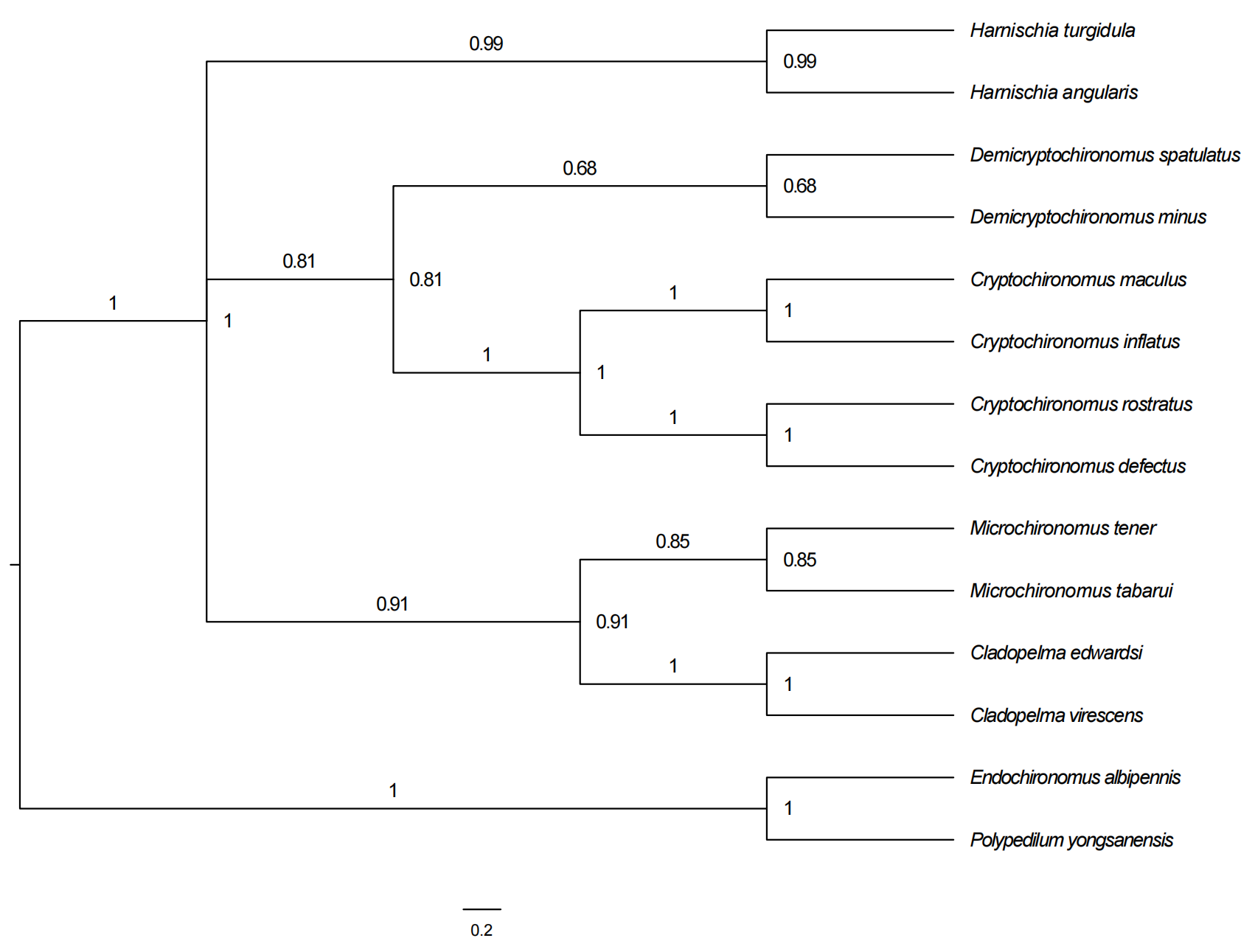

Supplement: Supplementary file 1 [file insects-15-00642-s001.zip › S15.tif]

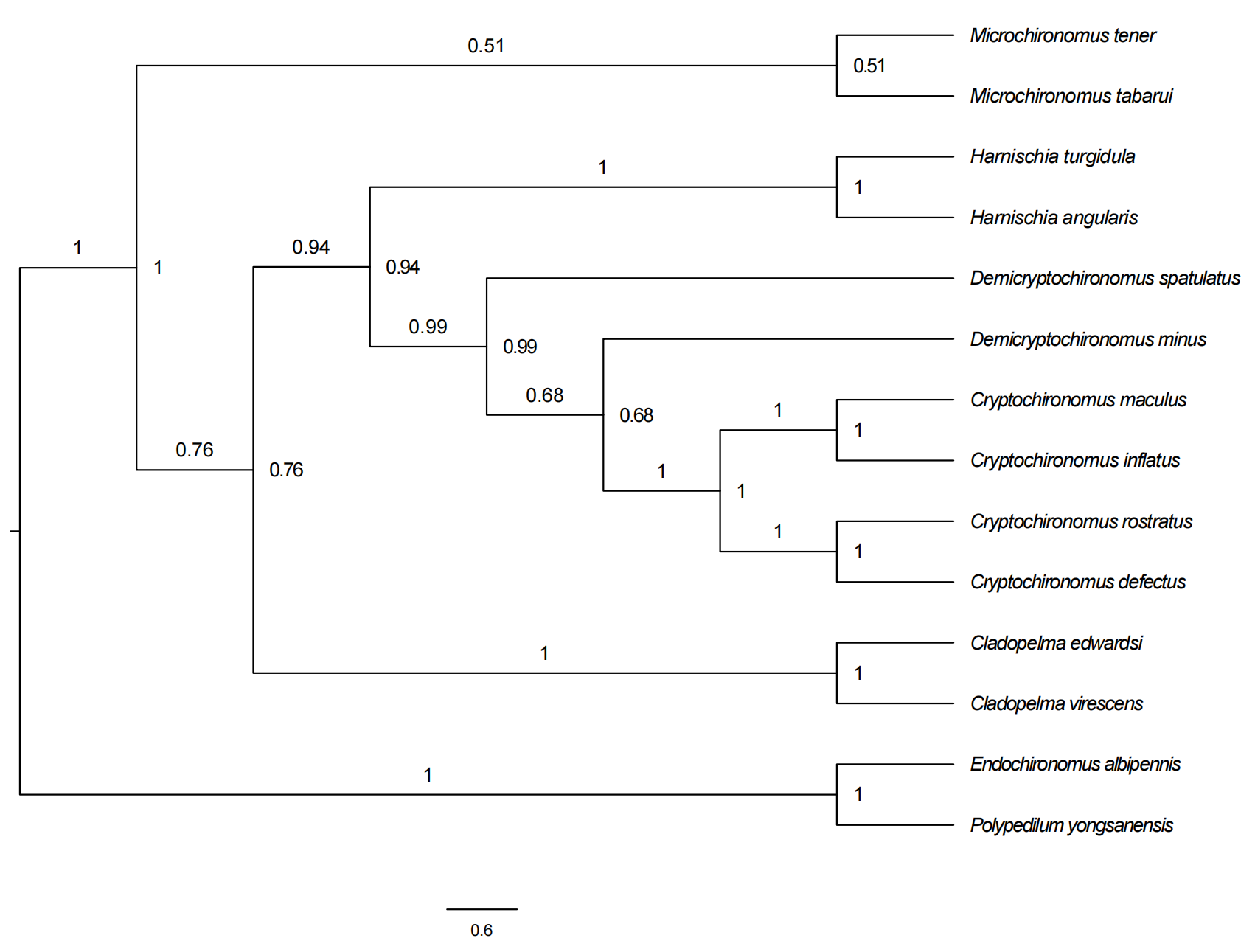

Supplement: Supplementary file 1 [file insects-15-00642-s001.zip › S16.tif]

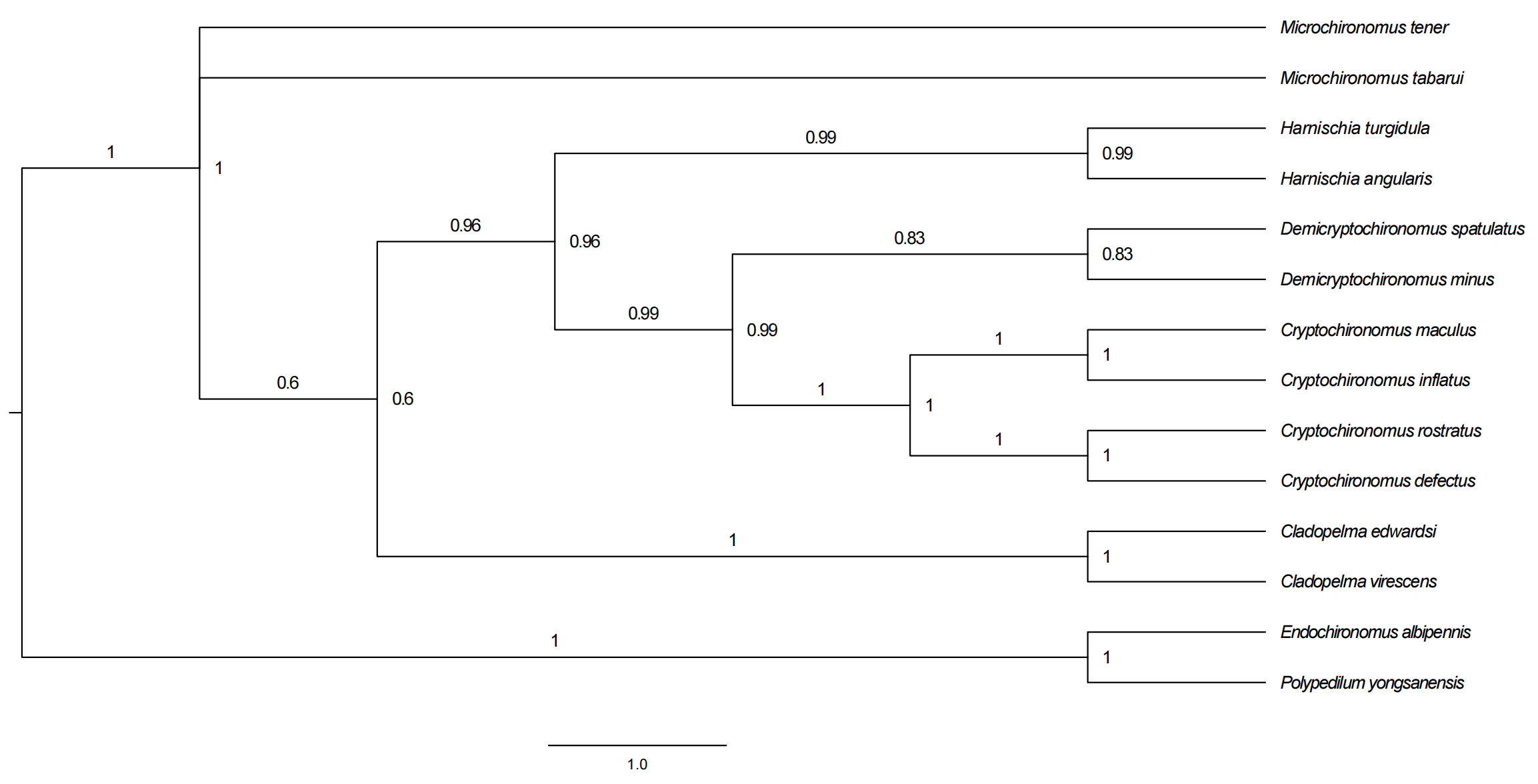

Supplement: Supplementary file 1 [file insects-15-00642-s001.zip › S17.tif]

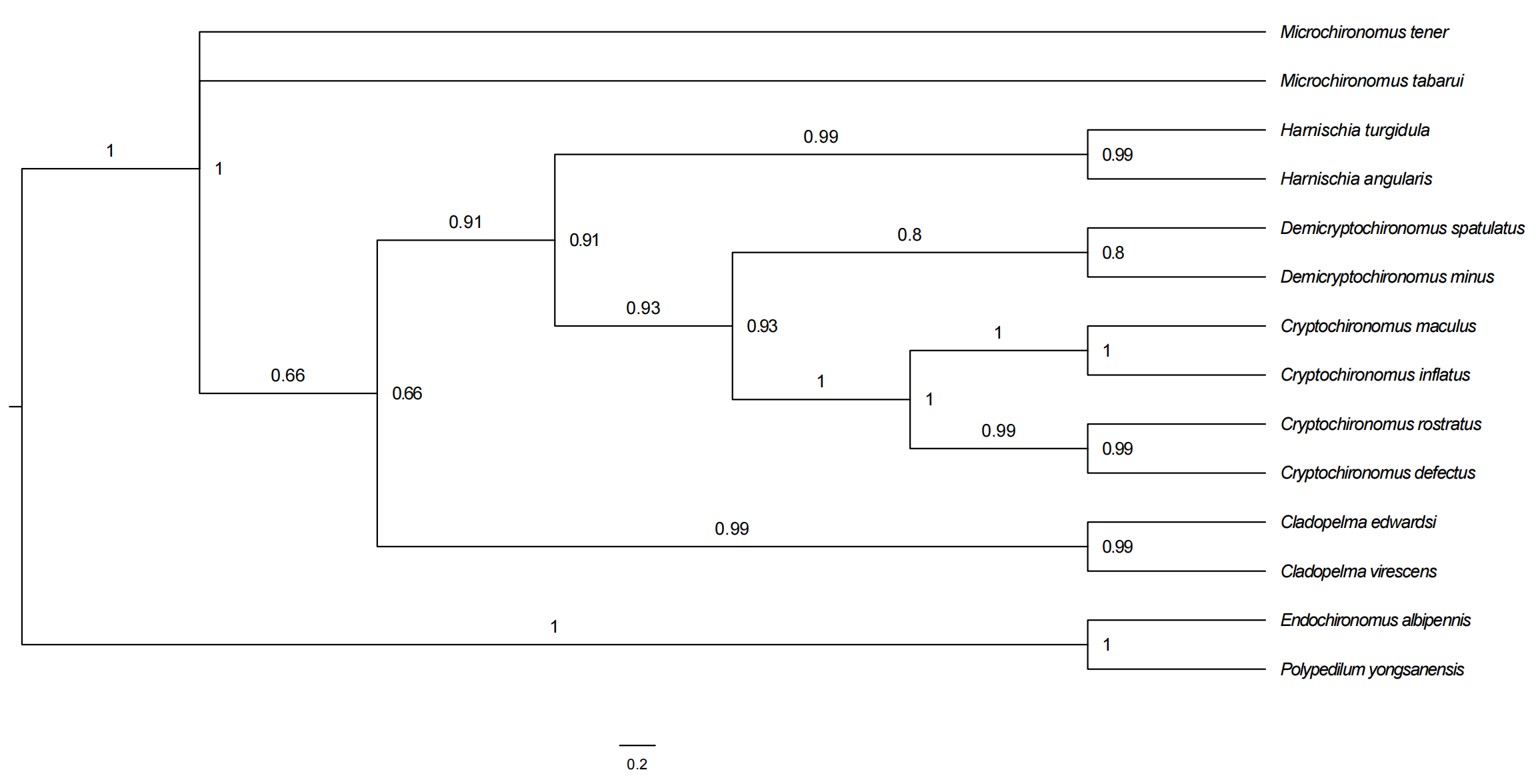

Supplement: Supplementary file 1 [file insects-15-00642-s001.zip › S18.tif]

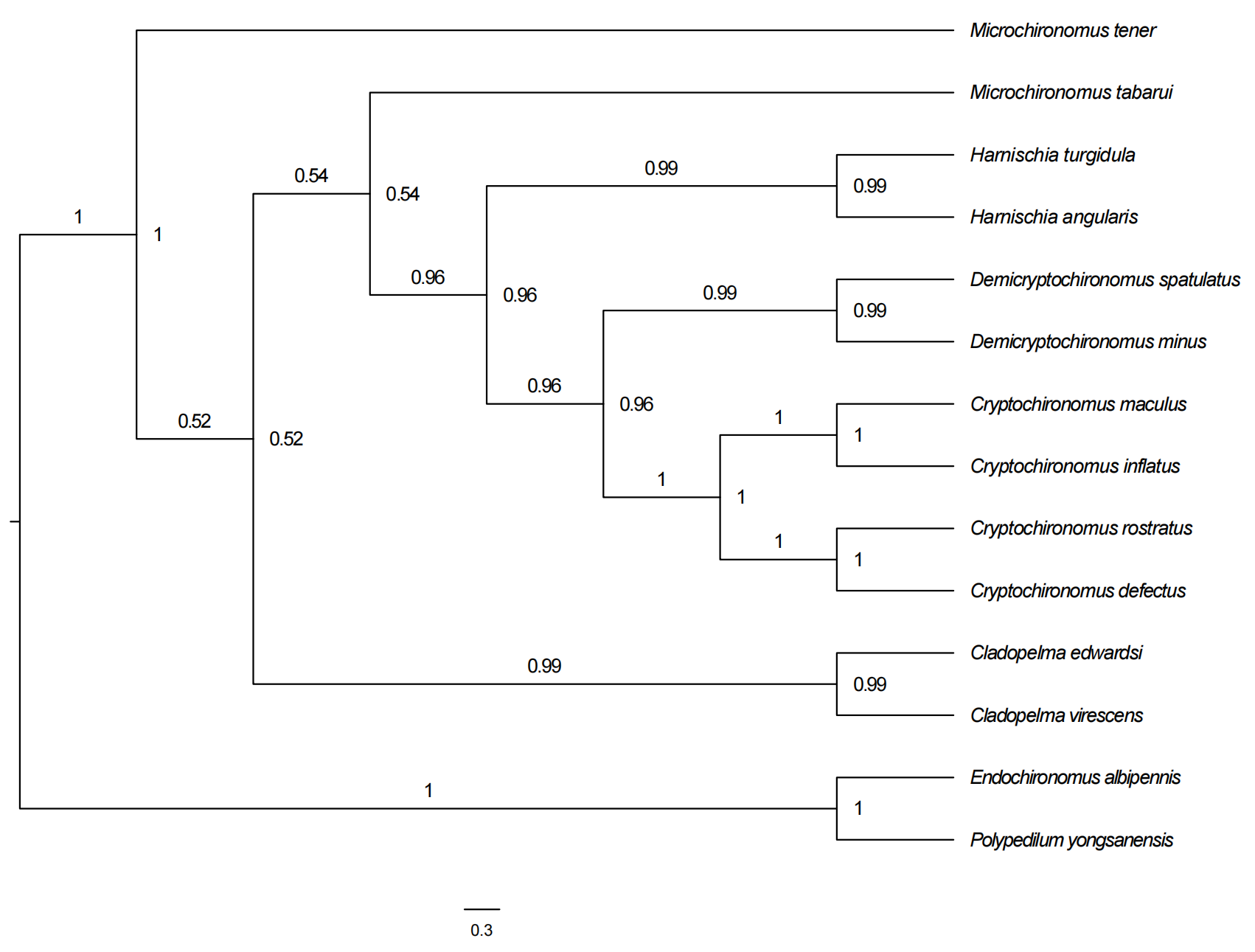

Supplement: Supplementary file 1 [file insects-15-00642-s001.zip › S19.tif]

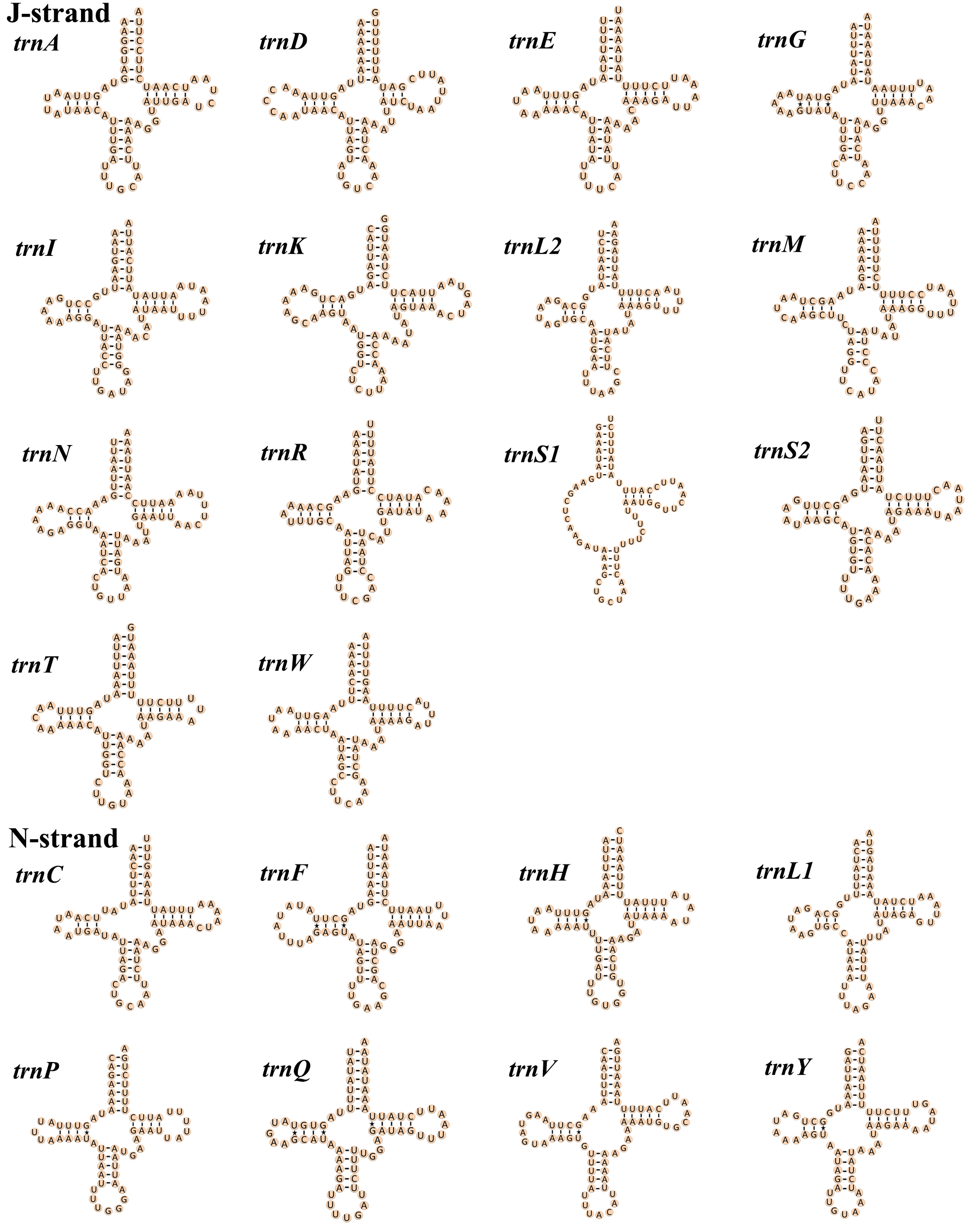

Supplement: Supplementary file 1 [file insects-15-00642-s001.zip › S2 Cryptochironomus sp..jpg]

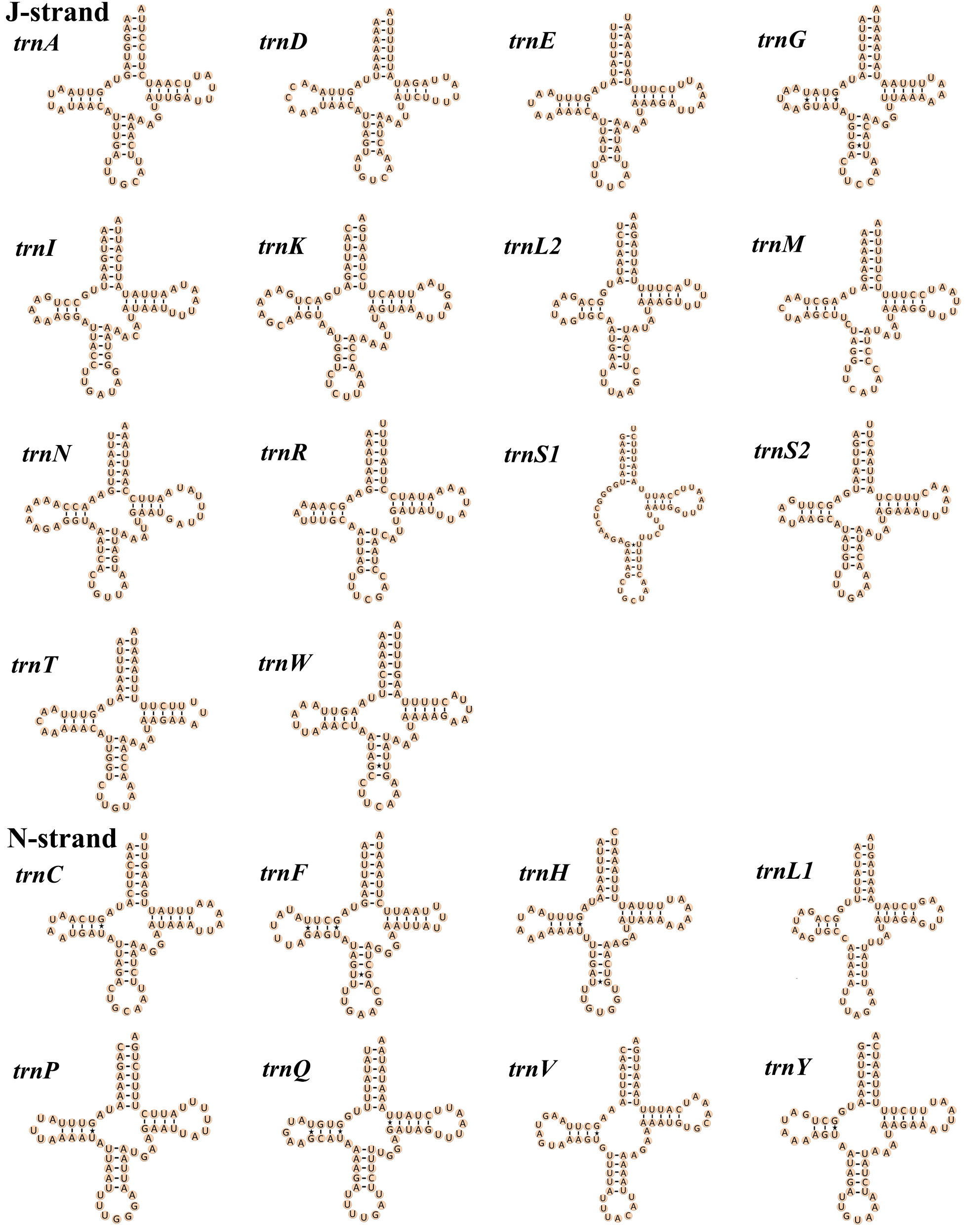

Supplement: Supplementary file 1 [file insects-15-00642-s001.zip › S3 Cryptochironomus defectus.jpg]

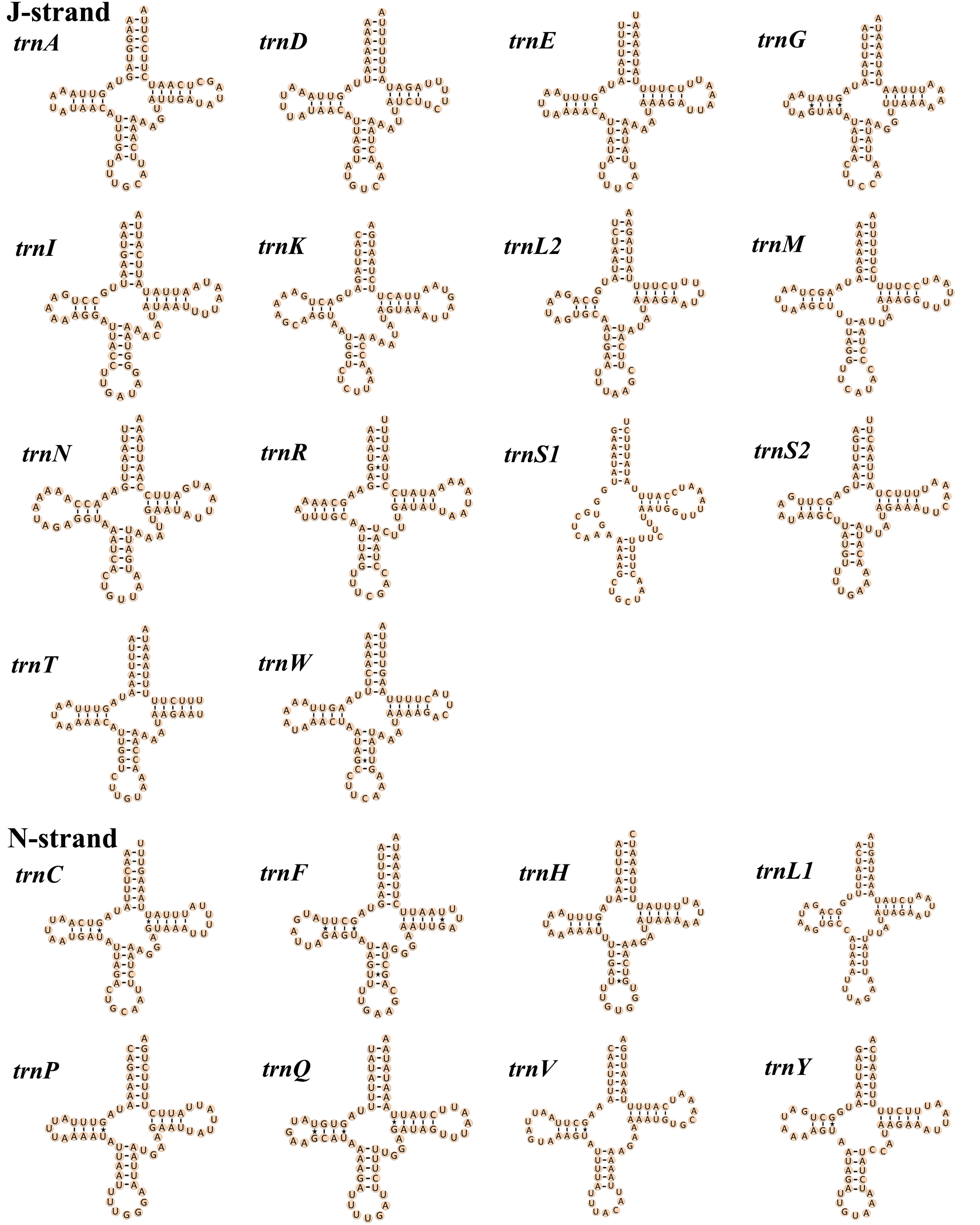

Supplement: Supplementary file 1 [file insects-15-00642-s001.zip › S4 Cryptochironomus rostratus.jpg]

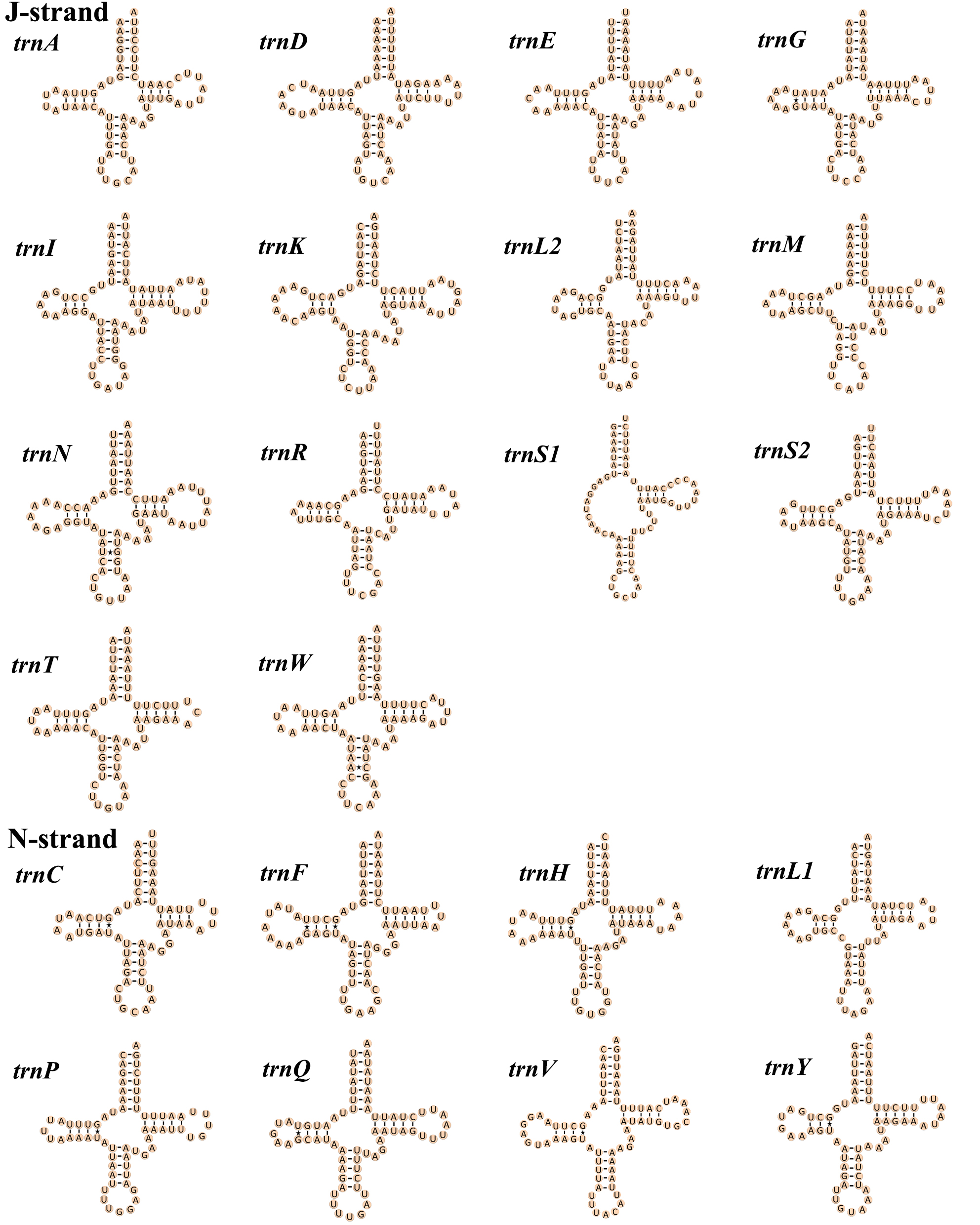

Supplement: Supplementary file 1 [file insects-15-00642-s001.zip › S5 Demicryptochironomus spatulatus.jpg]

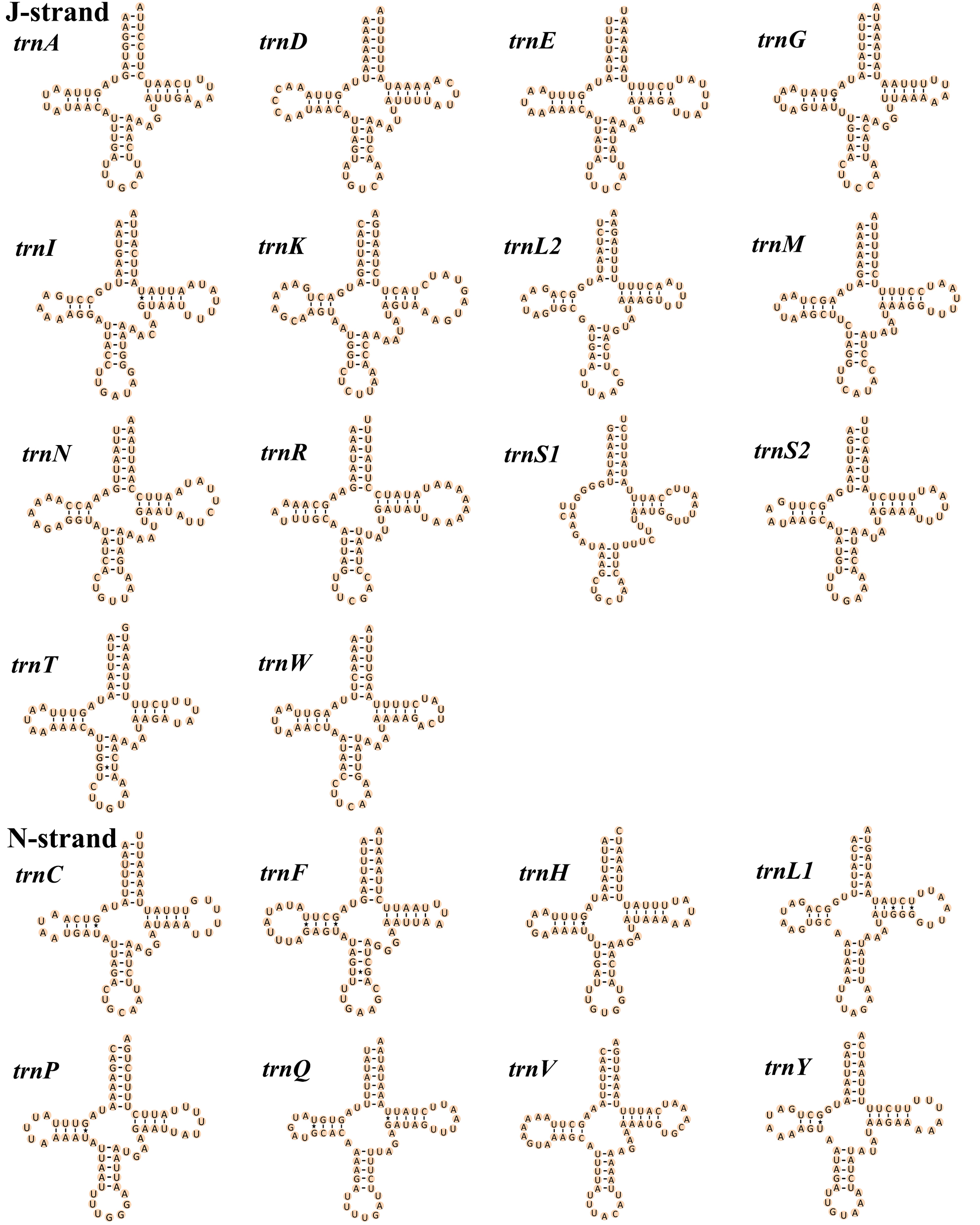

Supplement: Supplementary file 1 [file insects-15-00642-s001.zip › S6 Demicryptochironomus minus.jpg]

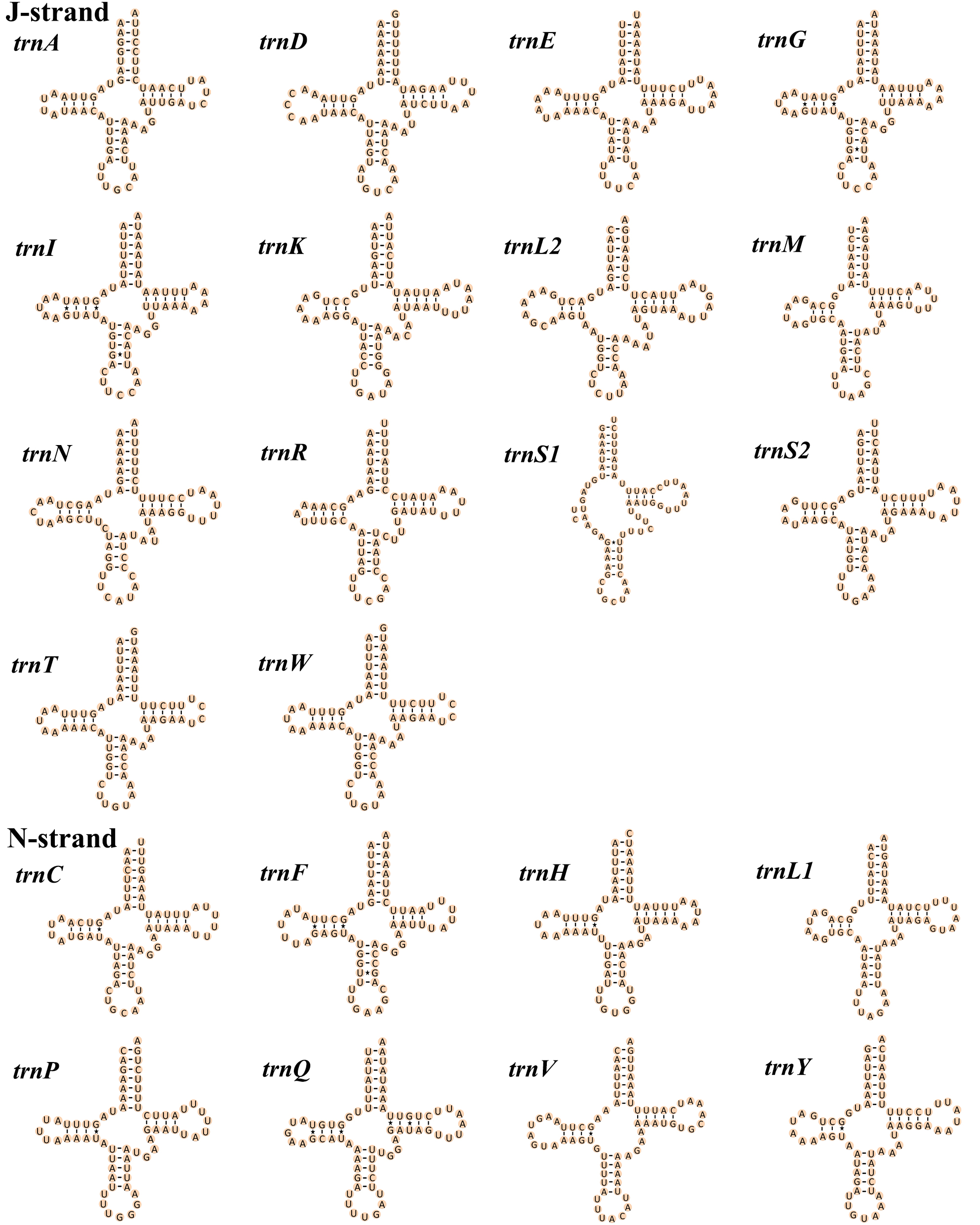

Supplement: Supplementary file 1 [file insects-15-00642-s001.zip › S7 Harnischia_angularis.jpg]

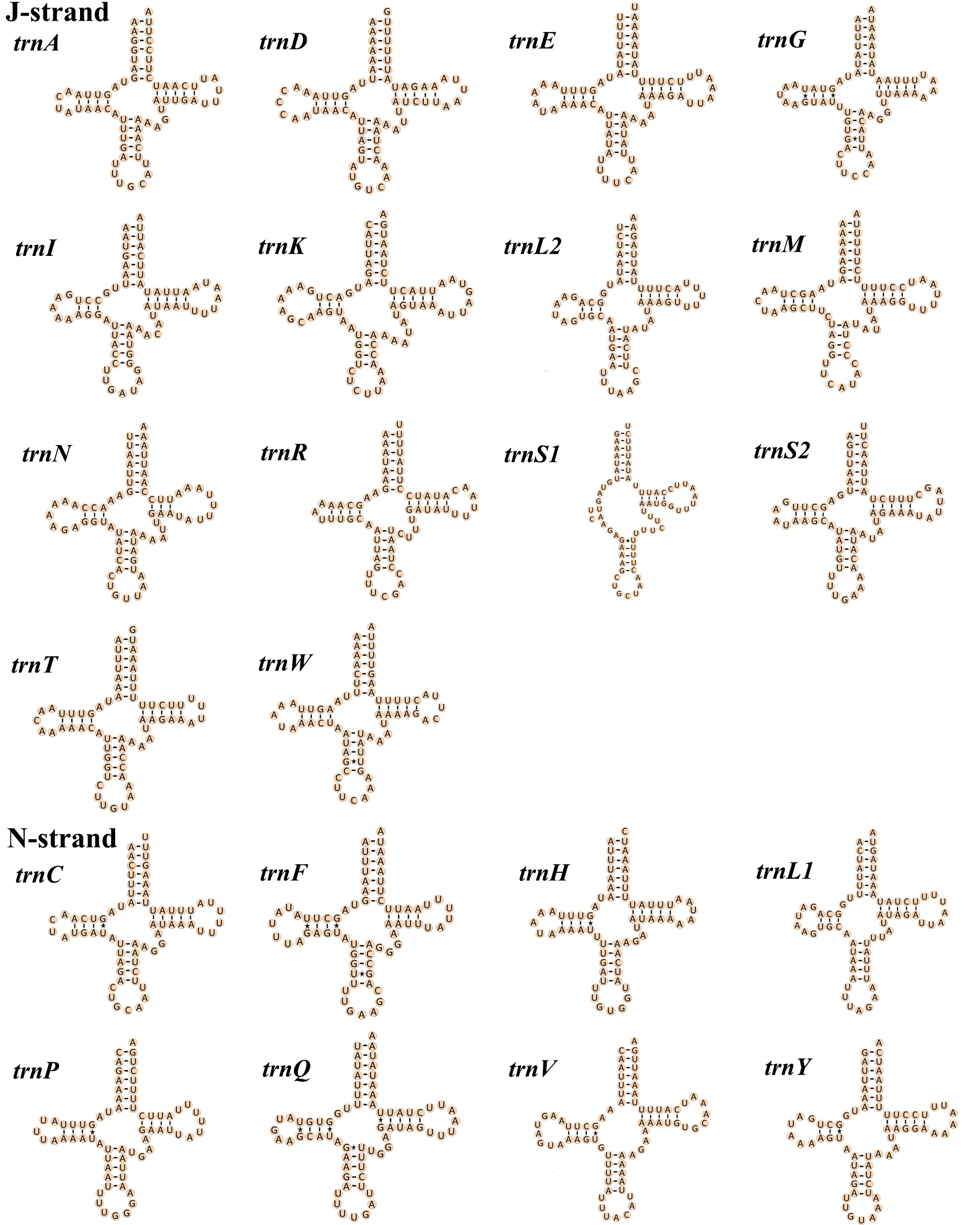

Supplement: Supplementary file 1 [file insects-15-00642-s001.zip › S8 Harnischia turgidula.jpg]

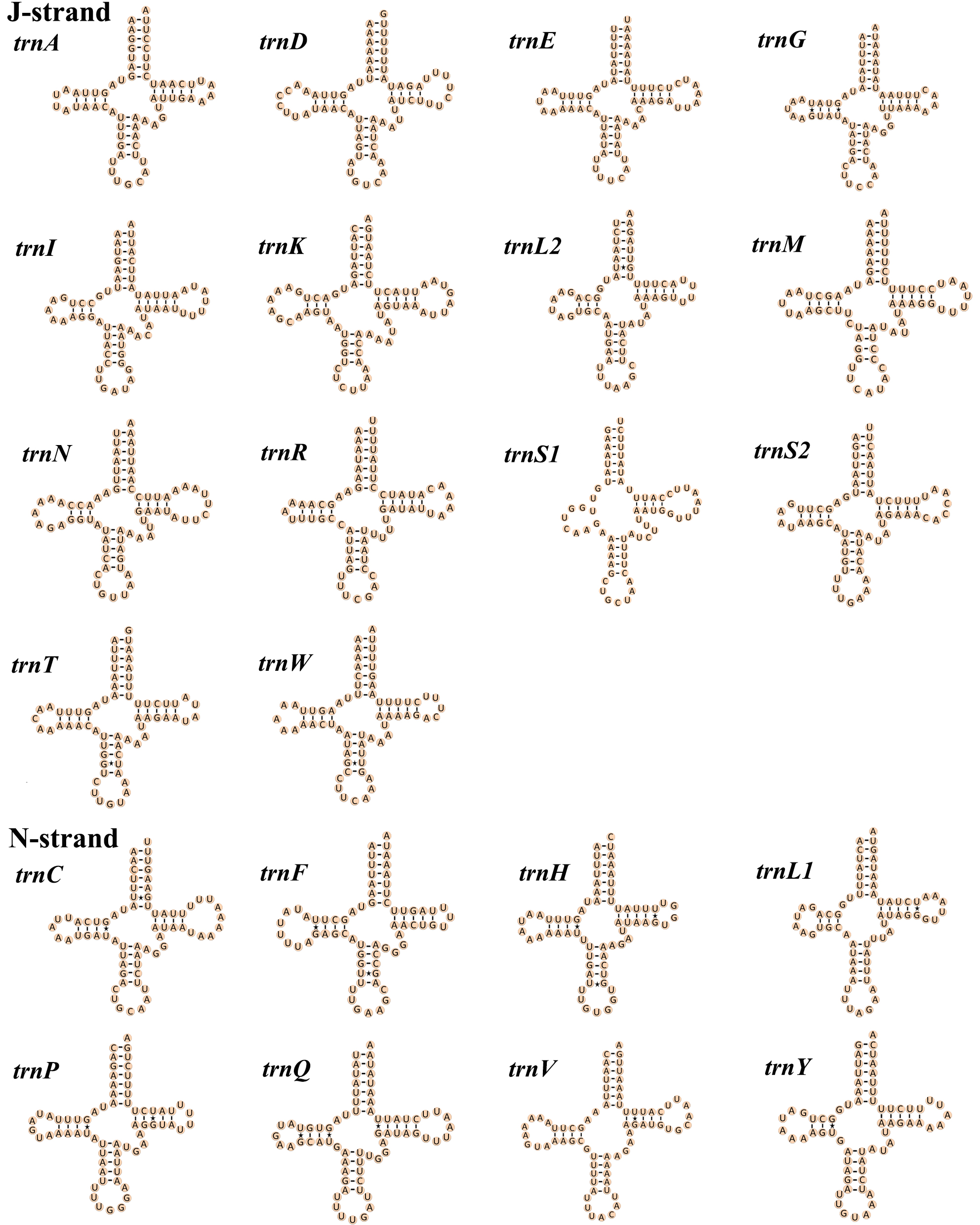

Supplement: Supplementary file 1 [file insects-15-00642-s001.zip › S9 Cladopelma edwardsi.jpg]
